# Macular Hole after Laser In Situ Keratomileusis in a 26-Year-Old Patient

**DOI:** 10.1155/2013/739474

**Published:** 2013-06-12

**Authors:** Guzel Bikbova, Toshiyuki Oshitari, Madoka Sakurai, Takayuki Baba, Shuichi Yamamoto

**Affiliations:** Department of Ophthalmology and Visual Science, Chiba University Graduate School of Medicine, Inohana 1-8-1, Chuo-ku, Chiba, Chiba 260-8670, Japan

## Abstract

The purpose of this study is to describe the 26-year-old patient with developed macular hole after bilateral laser in situ keratomileusis (LASIK). A macular hole with sharp margins and irregular surface of surrounding retina appeared in the left eye of the female 26-year-old patient two months after LASIK for correction of myopia (followup of 6 months). Although the best corrected visual acuity (BCVA) after LASIK was 1.0, after the macular hole has developed BCVA became 0.5. After surgery, the final visual acuity recovered to 0.7. Macular hole may develop after LASIK for myopia correction due to unknown changes of vitreoretinal interface. Complete informed consent must be obtained from patients with high myopic eyes before LASIK.

## 1. Introduction

Laser-assisted in situ keratomileusis (LASIK) is now a widespread method for myopia correction due to the lack of postoperative pain, lower incidence of corneal haze, and scarring, favorable, and rapid improvement of visual outcome for eyes with more than −6 diopters [1, 2]. Although several clinical studies had demonstrated the efficacy and predictability of LASIK, vitreoretinal complications including retinal tears, retinal detachments, retinal hemorrhages, macular holes, and choroidal neovascular membranes had been reported [3–8].

Several publications describe the developed macular hole after LASIK. Chan and Lawrence II have reported three eyes with developed macular hole after LASIK and photorefractive keratectomy in patients aged more than 30 years [8]. Ruiz-Moreno et al. reported the case of macular hole in a 53-year-old patient [9]. Arevalo et al. reported their study of 14 patients with full thickness macular hole after LASIK which was treated by vitrectomy [10]. The mean age was 45.5 years old. In this paper, we describe a case of developed full thickness macular hole after LASIK for the correction of myopia in a 26-year-old patient. We also alarm to obtain the complete informed consent before LASIK from the patients with high myopia because LASIK for myopia correction is still off-label use in Japan.

## 2. Case Report

**Figure 1:**
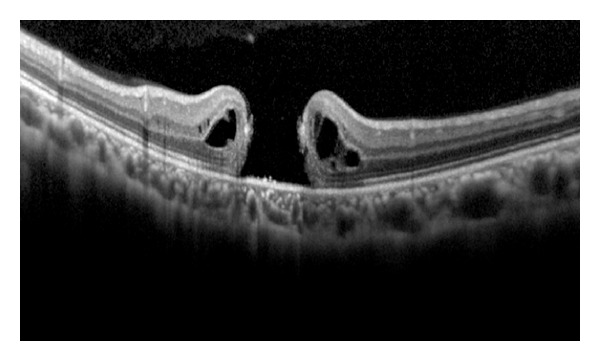
Optical coherence tomography (OCT) image showing full thickness macular hole with sharp margins and irregular surface of surrounding retina.

**Figure 2:**
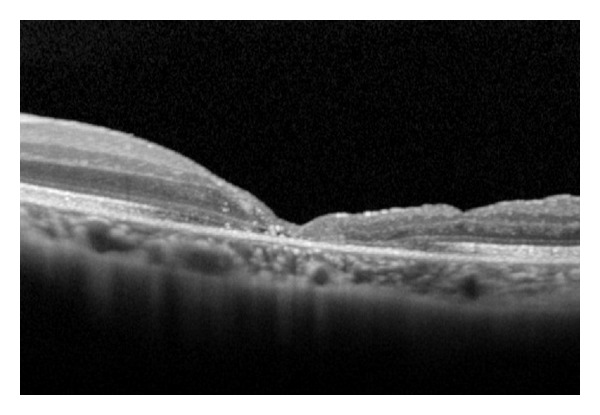
OCT after vitrectomy reveals a closed macular hole with a BCVA of 0.7.

A 26-year-old female with −7.0 D of spherical equivalent of refraction (SE) in the right eye and −7.25 D SE in the left eye underwent bilateral LASIK in August 2011. Two months after surgery, she noticed the blurred vision in the left eye. The best-corrected visual acuity (BCVA) was 0.5. Retinal examination revealed a stage 4 macular hole in the left eye. OCT images showed full thickness macular hole with sharp margins and irregular surface of surrounding retina (Figure 1). The patient underwent pars plana vitrectomy with internal limiting membrane peeling and intraocular gas tamponade to repair the macular hole (Figure 2). BCVA recovered to 0.7 3 months after surgery. Fundus examination revealed closure of the macular hole with mild retinal pigment epithelial changes in the central macula.

## 3. Discussion

According to the study of Morita et al. [11] and Patel et al. [12], macular hole tends to develop in young patients and also may be associated with a retinal detachment surrounding the macular hole.

The pathogenesis of macular hole development after LASIK remains unclear. Chan and Lawrence II [8] noted that LASIK surgery may introduce postoperative vitreoretinal interface changes. The significant increase of intraocular pressure during application of suction ring leads to the mechanical stretch of vitreum, and also excimer laser waves may induce vitreoretinal traction [11, 12]. Myopia is a risk factor for the macular hole development [13]. It may be that vitreoretinal interface changing after LASIK in predisposed myopic eyes leads to the macular hole development. 

In that way OCT screening may be helpful for the detection of the risk factor for the macular hole development [14]. A stage 0 macular hole based on OCT observations of vitreoretinal interface of patients with unilateral idiopathic macular hole was suggested by Chan et al. [15]. In the retrospective study of 94 patients with unilateral macular hole, it was found that in 28.7% of clinically normal fellow eyes abnormality of vitreoretinal interface with normal fovea anatomy was detected by OCT. Eyes with severe and moderate vitreoretinal abnormalities seemed to share characteristic features on OCT that increased their risk of macular hole development (stage 0 macular hole). Barak et al. introduced the mathematical analysis of anatomic foveal configurations predisposed to the development of macular hole [16]. They noted that suspicious macular configuration is easy to recognize on OCT scans and prehole macular hole fovea configuration is significantly different from normal foveal configuration.

In this case OCT was not performed before LASIK, that is why it is hard to determine if this patient was predisposed for the macular hole formation. LASIK for myopia correction is still off-label use in Japan. The Japanese guideline of LASIK alarms that over −6 D correction needs to obtain the complete informed consent and should be undergone with some medical reasons. Thus, further investigations of high myopic patients including pre- and post-LASIK OCT studies for determining the changes of vitreoretinal interface are needed.
